# Selenium: An Element of Life Essential for Thyroid Function

**DOI:** 10.3390/molecules26237084

**Published:** 2021-11-23

**Authors:** Francesca Gorini, Laura Sabatino, Alessandro Pingitore, Cristina Vassalle

**Affiliations:** 1 Institute of Clinical Physiology, National Research Council (CNR), 56124 Pisa, Italy; fgorini@ifc.cnr.it (F.G.); laura.sabatino@ifc.cnr.it (L.S.); pingi@ifc.cnr.it (A.P.); 2 Fondazione CNR-Regione Toscana Gabriele Monasterio, 56124 Pisa, Italy

**Keywords:** selenium, thyroid, deiodinases, Se-proteins, COVID-19

## Abstract

Selenium (Se), a microelement essential for life, is critical for homeostasis of several critical functions, such as those related to immune–endocrine function and signaling transduction pathways. In particular, Se is critical for the function of the thyroid, and it is particularly abundant in this gland. Unfortunately, Se deficiency is a very common condition worldwide. Supplementation is possible, but as Se has a narrow safety level, toxic levels are close to those normally required for a correct need. Thus, whether the obtaining of optimal selenium concentration is desirable, the risk of dangerous concentrations must be equally excluded. This review addressed the contribution by environment and food intake on Se circulating levels (e.g., geographical factors, such as soil concentration and climate, and different quantities in food, such as nuts, cereals, eggs, meat and fish) and effects related to its deficiency or excess, together with the role of selenium and selenoproteins in the thyroid pathophysiology (e.g., Hashimoto’s thyroiditis and Graves’ disease).

## 1. Introduction

Selenium (Se) is an essential micronutrient, necessary for the maintenance of different cellular functions (e.g., immune–endocrine function, signaling transduction pathways), although it is toxic like elemental Se as well as Se salts (even in small doses) [1]. The thyroid gland is particularly rich in Se, which takes part in the structure of antioxidant enzymes (e.g., glutathione peroxidase—GPx—and thioredoxin reductase—TrxR—as well as the three deiodinases—D1, D2, D3) [2]. Specifically, these proteins retain a key role in hormone metabolism and a powerful antioxidant activity directed against free radicals generated during the production of thyroid hormones (THs) [2]. The main source of Se is food, although its content varies in different human populations according to many factors (e.g., geographical characteristics, such as soil concentration and climate, and different content in food, such as nuts, cereals, eggs, meat and fish). Nonetheless, epidemiological data have demonstrated that Se deficiency characterizes a large percentage of individuals all over the world [3]. Moreover, circulating levels of this element have a narrow safety level, and toxic levels are close to those normally required for a correct need [4]. In fact, an excessive Se concentration may cause endocrine alterations in the synthesis of THs or increase the risk of Type 2 diabetes (T2D) [3,5]. Thus, whether the maintenance of adequate Se levels is desirable for all individuals, the risk of high blood concentrations (e.g., through excessive supplementation or a diet with a high Se content), which could be equally harmful, must be avoided. In this review, we discuss the sources of Se (the contribution by the environment and food intake) and consequences related to its deficiency or excess in the body. Then, the contribution of Se and selenoproteins in the thyroid physiology and their role in pathological thyroid diseases (e.g., Hashimoto’s thyroiditis and Graves’ disease) will be discussed.

## 2. Selenium in the Environment

Se is a metalloid with intermediate properties between a metal and a nonmetal [6], and at ordinary temperature it is a solid substance that occurs in the Earth’s crust as selenite, selenate and selenides associated with sulfide minerals in concentrations between 0.05 and 0.09 mg/kg [7,8]. Se can be detected in a wide array of environmental matrices including soil, air, water, plants and foods [9]. The Se content in soil depends on multiple variables such as soil type and texture, quantity of organic matter, rainfall amount and atmospheric deposition, and it generally ranges from 0.01 to 2 mg/kg, with a mean of 0.4 mg/kg [10]. While certain mountainous countries such as Finland, Sweden and Scotland are Se deficient (<0.1 mg/kg), a concentration of Se of at least >0.5 and up to 1200 mg/Kg is typical of alkaline soils developed from shales, the so-called seleniferous soils, which are widespread in dry regions of the United States, Canada, South America, China and Russia [1,11]. It is important to note that Se concentration in soil is not a strict indicator of plant absorption [8]. Soil Se is mainly inorganic, but it can also be bound to or incorporated in colloidal-sized organic matter [12]. Furthermore, Se mobility and, consequently, plant uptake, are positively correlated with soil pH, and this relationship is influenced by other factors such as redox conditions, clay content, ionic composition, microbial community and climatic setting [13,14,15]. In plants, Se and sulfur follow the same pathways in their uptake and metabolism and, importantly, organic Se (selenate and selenite) is taken at much higher rates (up to 100-fold greater) than inorganic species [16,17]. In particular, selenate is more water soluble than selenite and the most prevalent form in alkaline soils [18,19]. Currently, there is no evidence that Se is an essential micronutrient for higher plants; however, some plant species native to seleniferous soils accumulate Se to levels typically 100-fold higher than other vegetation and can store the metalloid at concentrations of 1000–15,000 mg/kg dry weight [10]. Se hyperaccumulators include approximately 30 species in the families of Brassicaceae (Stanleya), Fabaceae (Astragalus) and Asteraceae (Xylorhiza, Oonopsis, Symphyotrichum) [10]. They safely accumulate Se as methyl-selenocysteine and methyl-selenomethionine, which are forms that do not lead to oxidative stress and cannot be incorporated into proteins, within epidermal vacuoles of young leaves and reproductive organs [16,19,20]. Conversely, non-accumulator plants commonly contain <100 mg Se/kg dry weight when growing on seleniferous soils and mainly store Se in vascular tissues in leaves [11,19]. Both natural emissions (e.g., crustal weathering, sea spray, volcanic eruptions and biological activity in the marine and continental biosphere) and human activities (e.g., fossil fuel combustion, non-ferrous metal production, manufacturing, use of agricultural products) contribute to atmospheric Se [10,21]. Anthropogenic Se pollution in the environment accounts for about 40% of the emissions of Se in the atmosphere and the aquatic system, which has prompted the US Environmental Protection Agency to set the regulatory discharge limit of 5 μg Se/L for industry [22]. At a global level, average Se concentration in freshwater is 0.02 µg/L, while in seawater it is below 0.08 µg/L [23]. In well or underground water, Se originates from both atmospheric deposits and soil drainage and, although in most cases it does not exceed 10 µg/L, Se levels may approach hundreds of µg/L as a consequence of increasing overuse of Se-based fertilizers [6,24,25]. Hence, in the last few decades, Se contamination in ground and surface water in numerous river basins worldwide has become a critical issue [26]. Moreover, concentration increases depending on pH as a result of conversion into compounds of greater solubility in water, such as selenites and selenates [27], which show a pro-oxidant action in the organisms and are considered very toxic in case of elevated intake [26]. In the European Union [28] and the United States [29], the upper Se limit and maximum contamination level in drinking water are 20 and 50 µg/L, respectively. On the other hand, the World Health Organization (WHO) sets a provisional guideline value for Se in drinking water at 40 µg/L [30]. Generally, most drinking waters contain Se at concentrations that are much lower than 10 µg/L and are, therefore, not generally considered as a nutritional source of the element [27,31]. However, geogenic sedimentary sources of Se have been identified as being responsible for Se contamination in some wells producing drinking water [32]. The atmosphere plays a fundamental role in the transport, transformation and fate of Se in the environment [21]. The most represented Se species in the atmosphere include particulate Se, volatile organic compounds (dimethyl selenide and dimethyl diselenide) and volatile inorganic forms (selenium dioxide, hydrogen selenide, elemental Se) that are, however, very unstable in the atmosphere and characterized by a short lifetime [6,21]. Natural background levels of atmospheric Se, mostly in the particulate form, are very low and range from 0.1 to 10 ng/m3 in urban areas but can reach higher values near copper smelters [27].

## 3. Selenium in Food and Intake

Although environmental and occupational exposure and smoking habits may contribute to exposure, the main source of Se is dietary intake [1,33]. The Se concentration in both plant and animal foodstuffs is largely influenced by geographical variations of Se content and bioavailability species in soil and water [6,34], utilization of Se-enriched fertilizers [1] and self-supplementation with Se [35]. Furthermore, dietary assessment is challenging due to the wide variability in the Se content of foods and the complex extraction methods that can potentially affect Se species, and thus food composition tables often provide imprecise estimates of Se intake [1,36]. In the diet, Se is present primarily as selenocysteine (SeCys) and selenomethionine (SeMet) and, in lower amounts, as the inorganic compounds selenate and selenite [36]. Cereals are the major dietary sources of Se in most countries, as they tend to be consumed in large amounts, followed by meat, fish, eggs and dairy products [27,37]. SeMet is predominant in bread and cereals, such as wheat, other grains and soya, with Se concentration varying from 0.001 to 30 µg/g, and in Brazil nuts, which rank at the top of the ten richest food products containing Se (range ~0.03–512 µg/g) [1,38]. The most represented forms in the Se-accumulating plants Allium (garlic, onion, leek and wild leek) and Brassica (rapeseed, broccoli, cabbage) families are Se-methyl-selenocysteine and γ-glutamyl-Se-methyl-selenocysteine (considered “detoxification agents”), while SeMet and selenate, plus smaller amounts of SeCys, are in non-Se-accumulating plant foods [1,39]. Of note, both Se organic forms can be misincorporated in proteins in place of methionine and cysteine, respectively, leading to abnormal and potentially toxic products if in excess [40]. Other vegetables (e.g., carrots, peas, beans, potatoes, tomatoes) contain a maximum of 6 µg/g Se and, similarly, fruits rarely exceed a Se content of 10 µg/g [8]. The main Se species in animal foods are SeMet and SeCys [39], while the Se content of foods from animal sources varies according to the feed used and whether it is supplemented with inorganic or organic Se [8]. In omnivorous people, meat and fish represent the largest proportions of Se intake [8]. Se concentration is relatively high in offal, from heart, kidney and liver, from beef (range 0.55–4.5 µg/g) [41] and, for fish, in cod, shark and canned tuna (1.5, 2.0 and 5.6 µg/g, respectively) [1]. A whole egg has an average Se content of 15 µg/g, while a cup of milk or yogurt contains approximately 8 µg/g Se [42], with organic forms and selenite as Se predominant species [1]. Because Se is an essential element for humans but, contemporarily, an excessive consumption can result in toxic effects, various national and international organizations have established both reference values and upper limits for Se exposure [27]. Se deficiency in humans occurs when dietary intake is lower than 40 µg/day, whereas toxicity can be observed at daily levels above 400 µg [24]. Individual Se intake shows a large variability across countries, ranging from 3 to 7000 µg/day, with the highest values observed in Venezuela as well as in parts of China and North America [1]. In the US, Se intake ranges from 60–220 μg/day, whereas lower values have been reported in Europe, with 30–90 μg/day recorded in Western and Central Europe and low or deficient intakes (7–30 μg/day) in Eastern European countries [43]. Various biomarkers of intake or status can be used, in particular plasma/serum Se levels, which reflect exposure up to a few days and weeks and also allow speciation analysis [36,44]. Another approach to evaluate Se exposure is based on measuring plasma levels of selenoproteins, namely proteins containing at least one SeCys and primarily involved in antioxidant actions, such as Se-dependent glutathione peroxidase GPx1 and selenoprotein P (SEPP1) [45,46,47]. The WHO sets the recommended nutrient intake for Se at the level of 26 μg/day for females and 34 μg/day for males, in both cases between the ages of 19 and 65, considering that two thirds of plasma saturation activity of GPx would be achieved after intakes of 27 μg/day in males weighing 65 kg [48]. On the other hand, assuming that a plasma Se concentration below 70 μg/L is associated with levels of GPx activity and selenoprotein P concentration that have not reached a maximum level, other agencies have established Se reference values from 50 to 70 μg/day for adults and lower levels for younger people depending on age [36,47,49,50]. Se supplementation, available both in multivitamin/multimineral supplements and a stand-alone product, often in the forms of SeMet or of Se-enriched yeast (grown in a high-Se medium) containing a mixture of different Se species, may represent a valuable tool in Se-deficiency regions, providing an average additional intake of 5–30 µg/day [1,42,51]. Besides, various biofortification strategies have been developed to increase the Se content in edible parts of agricultural products [51]: (1) use of selenate- or selenite-based fertilizers for the soil [52]; (2) plant breeding in order to select varieties with high Se accumulation capacity [53]; and (3) foliar application of selenite and selenate to cereals and vegetables to increase Se concentration, as well as amino acids, phenols, anthocyanins, sugar and antioxidant activity [54]. Notably, agronomic Se biofortification has many advantages if compared to direct Se supplementation, as inorganic Se absorbed by plants is transformed into organic forms, which present a higher bioavailability [54].

## 4. Selenium and Thyroid Hormone Deiodinases

**Figure 1:**
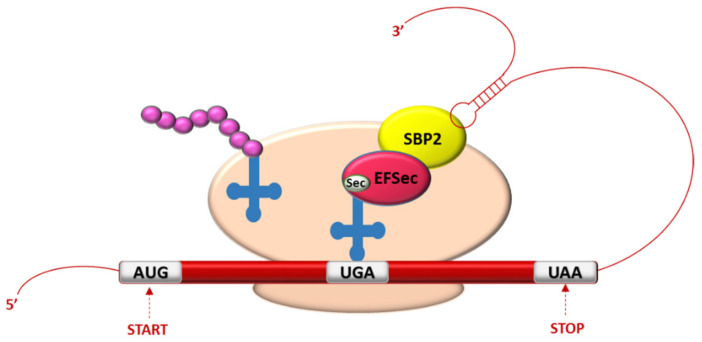
Schematic representation of selenocysteine (SeCys) insertion and the main involved factors. UGA is the SeCys-specific codon. SECIS binding protein 2 (SBP2) is associated to ribosomes and binds the SECIS element. SeCys-specific translation elongation factor (eEFSeCys) interacts with SeCys-tRNA and allows the incorporation of SeCys in the elongating polypeptide.

The thyroid is the organ with the highest content of selenium per tissue unit and it accumulates in the selenoproteins where it is present as SeCys [55]. SeCys is encoded by a UGA codon, which is normally considered a stop codon for translation. During protein translation, the signal driving the SeCys insertion is the presence of a stem-loop mRNA structure located in the 3′-untranslated region, called “SeCys insertion sequence” (SECIS) [56]. During the translation, when a ribosome encounters the UGA codon, SECIS element allows the re-coding of UGA as SeCys-specific codon and, in the process, two key factors are involved: the SECIS binding protein 2 (SBP2), which is associated to ribosomes and stably binds the SECIS element, and the SeCys-specific translation elongation factor (eEFSeCys), which interacts with SeCys-tRNA and permits the incorporation of SeCys in the elongating polypeptide (Figure 1) [57].

The first identified selenoproteins were the GPx, which protect the cells against oxygen free radicals damage [58]; TrxR, considered a powerful antioxidant compound that is also involved in the regulation of several transcription factors (i.e., NF-Kb, Ref-1, P53); and the three deiodinase isoforms (D1, D2 and D3) that are strictly linked to TH regulation and are located in virtually all tissues in the organism [46]. Deiodinases are 29–33 kDa integral membrane selenoproteins, formed by a single transmembrane domain, and they form dimeric structures. SeCys is located in the active site of the three enzymes, in the N-terminal part of the molecule and is crucial for nucleophilic attack occurring during the deiodination [59]. Interestingly, D2 has a second SeCys in the C-terminal part of the protein but its function is still mostly unknown, even though it is not critical for enzymatic activity [60]. The three enzymes have different cellular locations, depending on their functional role, as D1 and D3 are at the plasma membrane level, whereas D2 is at the endoplasmic reticulum membrane [61]. Thyroxine (T4) is the principal TH produced by the thyroid and is converted in triiodothyronine (T3) by D1 and D2 by outer ring deiodination of T4 molecules. T3 is considered the biologically active hormone, while T4 is mainly a prohormone. Both T3 and T4 can be inactivated by inner ring deiodination by D3 and, in minimal part, by D1, with the formation of catabolic inactive products reverse T3 (rT3) and T2, respectively [62]. In physiological conditions, the role of the three enzymes is to maintain the homeostasis of THs and their activity in the body. In particular, D1 is responsible for regulation of TH levels in the circulation, whereas D2 and D3 are involved in the local control of intracellular T3 concentration [63]. Virtually all tissues in the body receive the same signal by circulating T3; however, biological response and T4 to T3 conversion mainly depends on D2 and D3 activities in the target tissues, in accord with the local metabolic request.

## 5. Selenium and Autoimmune Thyroiditis (AIT)

**Figure 2:**
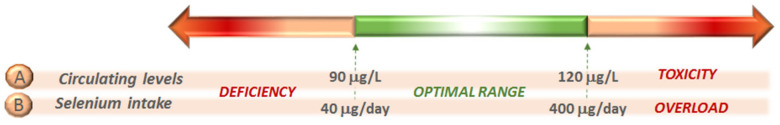
(**A**) Narrow range of adequate blood Se concentration, levels below and above, risk of deficiency and toxicity, respectively. (**B**) Recommended daily intake of Se, below and above, risk of adverse effects on health.

Autoimmune thyroiditis (AIT) occurs in about 0.3–1.5/1000 subjects/year, with a major frequency in middle-aged women more so than in men, and it affects up to 5% of the world population [64]. Graves’ disease (GD) and Hashimoto’s thyroiditis (HT) are the most common forms of AIT. A third of GD patients develop ophthalmic signs of GD. It is known that patients with thyroid disease (including hypothyroidism, subclinical hypothyroidism, autoimmune thyroiditis and enlarged thyroid) have reduced Se levels [65]. Thus, many trials, especially those conducted in geographical areas with diffuse low or borderline Se status in the population, aimed to assess whether Se supplementation may affect the evolution of thyroid immune diseases [66,67]. Overall, available studies suggest that Se supplementation may induce a decrease in circulating thyroid autoantibodies. However, since data are inconsistent due to patient number heterogeneity, different forms of Se supplements, duration of the supplementation, instrumental evaluation of thyroid function and serum Se measurement, they may not demonstrate a definitive relationship [67]. Moreover, possible interventions could be focused on patients residing in areas with low Se availability or with low- or sub-optimal Se levels, which can particularly benefit from this supplementation, avoiding adverse consequences associated with a high Se status. This is particularly relevant because the range of adequate serum Se is narrow (about 90–120 µg/L, estimated according to GPx activity and selenoprotein blood concentration), and below and above this interval there is the risk of deficiency and toxicity, respectively, with possible adverse effects on health (Figure 2) [50].

### 5.1. Grave’s Disease (GD)

GD is an autoimmune disease characterized by the production of thyroid-stimulating immunoglobulin, having similar effects to the thyroid stimulating hormone (TSH) and by the consequent overproduction of THs. Patients with newly diagnosed GD presented low Se levels. In addition, GD disease remission (in subjects followed for 20.1 months) was associated with higher serum Se levels (>120 µg/L) that inversely correlated with TSH receptor autoantibodies (TRAb), suggesting beneficial effects of Se on the thyroidal autoimmune process and GD outcomes [68,69]. Accordingly, many studies have attempted to evaluate Se supplementation as co-treatment with traditional drugs in patients with GD [70]. Available data indicated that Se supplementation can enhance the effect of antithyroid drugs (e.g., methimazole) in patients with recurrent GD, lowering TRAb level towards normalization, although other studies did not find significant effects on either the clinical course or serological parameters (e.g., free triiodothyronine/free thyroxine ratio-fT3/fT4), TRAb, prevalence of moderate-to-severe Graves’ orbitopathy, thyroid volume, remission rate) [71,72,73,74]. In this context, a recent systematic review and meta-analysis including 10 randomized trials, characterized by a Se supplementation period of 3 or 6 months, revealed clinically and statistically significant effects on fT4, fT3, TSH and TRAb levels in patients with GD, while, conversely, the 9-month Se regime did not prove to be more effective [75]. These inconsistent and apparently contradictory results are possibly attributable to differences between studies, such as length of treatment period and follow-up, sample size, heterogeneity of patients, stage of disease and variability in Se status (often dependent on the geographical area). Furthermore, studies did not generally measure Se baseline levels, though Se supplementation provides no benefit if Se intake and serum level are adequate, whereas it is likely more effective in Se-deficient patients.

#### Graves’ Ophthalmopathy (GO)

Graves’ ophthalmopathy (GO), an autoimmune inflammatory disorder of the orbit and periorbital tissues (TSH receptor and TSH-receptor antibody culprit factors), is a common finding in Grave’s patients, more frequent in females than in males, with an estimated prevalence in Europe between 90 and 155/100,000 population [76]. Since GO subpopulation in GD presented lower Se levels than in subjects without orbitopathy, and severe Se deficiency is associated with more serious GD orbitopathy, the Se supplementation issue in patients with GO aroused great interest [77,78,79]. Some effects by which Se may influence GO are related to its antioxidant actions and include the reduction in proliferation and secretion of pro-inflammatory cytokines in orbital fibroblasts and the release of hyaluronic acid [80]. In light of the available results, the recommendation for Se use in mild cases (but not in moderate/severe) has been confirmed in the 2021 guidelines of the European Group On Graves’ Orbitopathy (EUGOGO) for sodium selenite (200 μg per day for 6 months), or SeMet (100 μg/day), to achieve a higher rate of improvement in both GO quality of life (QoL) and overall ophthalmic outcome, as well as a lower rate of progression towards more severe GO [81,82].

### 5.2. Hashimoto’s Thyroiditis (HT)

HT, also known as chronic lymphocytic thyroiditis, is an autoimmune disease characterized by autoreactive lymphocytes invading the thyroid gland and causing hypothyroidism. Some data indicate that Se levels are lower in patients with HT than in healthy subjects and are inversely related to TSH or antithyroid antibody levels [83,84]. In a study conducted in a Danish area with mild iodine deficiency, Se deficiency is associated with thyroid gland volume and nodule formation before and after introduction of iodine supplementation [85]. In addition, Se was reported to be significantly reduced in patients with nodular goiter [65,86]. Since current evidence suggests that Se deficiency is a risk factor for increased thyroid gland volume, hypothyroidism, HT and thyroid nodules, Se supplementation could reasonably be proposed in Se-deficient geographic areas in HT patients. For example, Se (100 µg/day for 6 months) significantly reduced the level of antithyroid peroxidase antibodies when administered in newly diagnosed and previously untreated HT patients with euthyroidism or subclinical hypothyroidism living in a Polish area with low Se status [87]. Moreover, administration of 200 μg/day Se yeast tablets for at least 6 months in HT patients improved thyroid autoantibodies and thyroid function by increasing the antioxidant activity [88]. The “SETI study” showed that short-course SeMet supplementation was associated with a normalization of serum TSH levels, which is maintained for 6 months after Se withdrawal in 50% of patients with subclinical hypothyroidism, due to chronic autoimmune thyroiditis [89]. Therefore, considering that low Se levels are related to an increased risk of developing antithyroid antibodies, and Se supplementation can reduce TPOAb titres, we can hypothesize that a reduction in the doses of levothyroxine necessary for hypothyroidism therapy and/or a prevention in the progression of subclinical hypothyroidism are feasible. Indeed, a meta-analysis published in 2016 reported that Se supplementation decreased levels of thyroid autoantibodies after 3, 6 and 12 months in an LT4-treated AIT population and after 3 months in an untreated AIT cohort [90]. However, not all studies observed an improvement of thyroid function after Se supplementation, especially those lasting over time [91]. Notably, two recent meta-analyses showed insufficient evidence for the clinical efficacy of Se supplementation for all patients with chronic AIT, and the same findings were reported by a previous Cochrane Database analysis of four randomized controlled studies on Se supplementation in HT subjects [92,93].

## 6. Other Pathophysiological Conditions beyond Intake Potentially Affecting Se Levels and the Relationship with Thyroid Function

Beyond the differences in Se intake, some other coexisting pathophysiological conditions, such as bacterial and viral infection or pregnancy, are associated with higher Se demands or greater Se loss. During pregnancy, the fetus accumulates Se, depleting the mother if Se status is not satisfied by an additional Se intake; this occurs also in the lactation period, with the risk of leaving the mother and baby deficient in Se [94]. When 200 μg/day SeMet were given to pregnant women, thyroid inflammatory activity decreased, and risk of postpartum thyroid dysfunction and permanent hypothyroidism were significantly reduced [95]. The “SERENA study”, a multicenter, randomized, double blind, placebo-controlled trial, has recently demonstrated the beneficial effects on autoantibody titer during pregnancy and on postpartum thyroiditis recurrence after supplementation with L-SeMet (83 μg/day) in women treated for the entire duration of pregnancy and for 6 months after delivery [96]. Of note, in order to avoid the risk of overdose, a shared consensus on cut-off levels for Se administration as well as on doses and periods of supplementation would be desirable, maybe in parallel to evaluation of circulating blood concentrations. In this context, the Guidelines of the American Thyroid Association for the diagnosis and management of thyroid disease during pregnancy and postpartum do not recommend Se supplementation during pregnancy, pending further data from randomized control trials to assess safety and efficacy of Se supplementation in pregnancy at high risk of adverse events (e.g., miscarriage, preeclampsia, preterm delivery, fetal death) [97].

**Figure 3:**
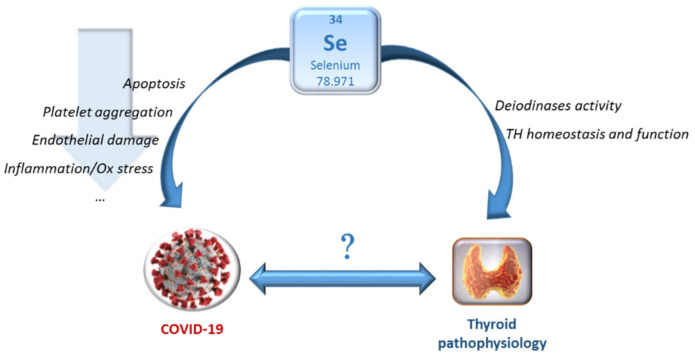
Selenium, Sars-CoV-2 and thyroid gland interconnection. Preliminary data suggest a possible bidirectional relationship between COVID-19 and thyroid dysfunction, and both conditions seem to be associated with Se deficiency (see text). Whether Se supplementation may modulate the relationship between SARS-CoV-2 infection and thyroid pathophysiology remains to be clarified in further studies. Abbreviation: TH—thyroid hormone.

It is known that Se deficiency is associated with greater susceptibility to viral RNA infections and more severe disease outcomes, whereas Se supplementation could benefit antioxidant capacity (GPx and TrxR) and reduce apoptosis, endothelium injury and platelet aggregation [98]. In particular, in this era of pandemic, the relationship between COVID-19 and Se is interesting. Se levels are lower in patients with COVID-19 than in healthy subjects [99,100]. Furthermore, Se deficiency has been associated with the risk of COVID-19 complications and mortality, although the relationship between Se and COVID-19 severity is not definitively demonstrated [101,102,103,104,105,106,107]. Besides, an association between soil Se content and COVID-19 incidence was found in different cities in Hubei Province, as well as an association between soil Se content and the reported outcomes of COVID-19 cases in China [108,109]. A German study suggested that 1.0 mg intravenous Se daily and different combinations of artificial nutrition (containing various amounts of Se and Zn) in critically ill patients with COVID-19-induced acute respiratory distress syndrome may reduce inflammatory biomarkers and improve the immune response [110]. Hence, also in this case, Se supplementation may have important consequences, and monitoring the Se status during the SARS-CoV-2 infection, in both mildly and severely affected patients, and verifying the possible beneficial effects of Se supplementation, can represent a further potential tool both in prevention and as adjuvant therapy for COVID-19. Interestingly, increasing data suggest a bidirectional relationship between COVID-19 and thyroid dysfunction (e.g., through effects on immune system and induction of the cytokine storm by Sars-CoV-2, as well as preliminary findings on adverse effects of preexisting thyroid disease on the prognosis of COVID-19) [111]. In this context, given the possible effect of COVID-19 on thyroid dysfunction and AIT, it will also be interesting to further investigate the association of these conditions with Se deficiency and to evaluate the possible effects of Se in modulating the relationship between SARS-CoV-2 infection and thyroid pathophysiology (Figure 3) [111,112,113,114].

## 7. Conclusions

Se is a trace mineral essential for life and a pillar for thyroid functioning, as an active component of thyroid selenoproteins. Some studies suggest the correlation between Se deficiency and autoimmune thyroid diseases as well as benefits of selenium supplementation in these conditions. Nonetheless, the range of optimal required plasma Se levels is quite narrow, and unambiguous relationships between Se intake, status and health needs to be further defined in the future.

Moreover, intriguing aspects of thyroid pathophysiology in relation to selenium have been evidenced in literature, opening future perspectives for a better understanding of thyroid biology:

(1) The Se uptake by the thyroid gland appears to be independent of SEPP1-mediated selenium supply utilized by organs such as the kidney and testis. This is exemplified by the knockout of SEPP1, which does not affect thyroid function, suggesting that the thyroid gland may be able to effectively accumulate, retain and recycle selenium, even in the absence of SEPP1 [115].
(2) Thyroidal Se content is typically not reflected by serum Se levels, as liver secreted SEPP1 is the main determinant of plasma Se levels [116]. Hence, thyroid tissue-specific markers of Se bioavailability and cellular action have yet to be identified.
(3) Rare mutations in the “SeCys insertion sequence binding protein 2” gene (SBP2), a protein required for selenoprotein synthesis, induce a multisystem condition that includes abnormalities in thyroid biomarkers (elevated serum fT4 and rT3 levels, low to low-normal serum T3 levels and normal to slightly elevated serum TSH levels, in the presence of reduced blood Se levels) [117]. In particular, low circulating levels of Se in patients with SBP2 mutations result from impaired synthesis of SEPP1 and GPx3, major carriers of Se in serum [117]. These interesting and rare cases, in addition to the SBP2 mouse model, support the role of SBP2 as a critical limiting factor in thyroid selenoproteins synthesis and, therefore, thyroid function [118].

## Abbreviations

AIT	autoimmune thyroiditis
Dx	deiodinase
fT3	free triiodothyronine
fT4	free thyroxine
GD	Graves’ disease
GO	Graves’ ophthalmopathy
GPx	glutathione peroxidase
HT	Hashimoto’s thyroiditis
rT3	reverse T3
Se	selenium
SECIS	SeCys insertion sequence
SeCys	selenocysteine
SeMet	selenomethionine
SEPP1	selenoprotein P
T2D	type 2 diabetes
T3	triiodothyronine
T4	thyroxine
TrxR	thyoredoxine reducatase
TSH	thyroid stimulating hormone
TRAb	thyroid stimulating hormone
